# The effect of toothpaste and thermal aging on extrinsically stained additively manufactured zirconia: A comparative study on surface roughness and stain durability

**DOI:** 10.1111/jopr.70160

**Published:** 2026-05-04

**Authors:** Cecilia Santos Galvao, Ediz Kale, Mustafa Borga Donmez, Özgür Ozan Tanrıkut, Adriana da Fonte Porto Carreiro, Richard Johannes Wierichs, Hanan Al‐Johani, Burak Yilmaz

**Affiliations:** ^1^ Department of Prosthodontics Federal University of Rio Grande do Norte (UFRN), Natal, Rio Grande do Norte Natal Brazil; ^2^ Department of Prosthodontics Faculty of Dentistry Mersin University Mersin Turkey; ^3^ Department of Periodontology Faculty of Dentistry Mustafa Kemal University Hatay Turkey; ^4^ Department of Prosthodontics Faculty of Dentistry Biruni University Istanbul Turkey; ^5^ Department of Reconstructive Dentistry and Gerodontology, School of Dental Medicine University of Bern Bern Switzerland; ^6^ Department of Dentistry Federal University of Rio Grande do Norte (UFRN), Natal Rio Grande do Norte Brazil; ^7^ Department of Operative Dentistry and Periodontology University Hospital Bonn, University of Bonn Bonn Germany; ^8^ Department of Restorative Dentistry Faculty of Dentistry King Abdulaziz University Jeddah Saudi Arabia; ^9^ Department of Restorative Preventive and Pediatric Dentistry School of Dental Medicine University of Bern Bern Switzerland; ^10^ Division of Restorative and Prosthetic Dentistry The Ohio State University College of Dentistry Columbus Ohio USA; ^11^ Department of Prosthodontics Faculty of Dentistry Ankara University Ankara Turkey

**Keywords:** additive manufacturing, extrinsic stain, toothpaste, zirconia

## Abstract

**Purpose:**

The purpose of this in vitro study was to compare the average surface roughness (*R*_a_) and stain durability of extrinsically stained additively manufactured zirconia, after brushing with different toothpastes and thermal cycling, with those of subtractively manufactured zirconia and lithium disilicate.

**Materials and Methods:**

Forty‐eight disk‐shaped specimens (Ø13 × 2 mm) were fabricated from an additively manufactured zirconia (INNI Cera BCM W1000, AM‐ZR), a subtractively manufactured zirconia (VITA YZ ST, SM‐ZR), and a subtractively manufactured lithium disilicate (UP.CAD HT, SM‐LD) (*N* = 16 per material). One surface of each specimen was externally stained (B4, VITA AKZENT Plus) and glazed (VITA AKZENT Plus Glaze LT), after which baseline *R*_a_ and color measurements were recorded with a laser microscope and a spectrophotometer, respectively. Specimens were then subjected to 5000 cycles of brushing with either regular (RT) or whitening (WT) toothpaste (*n* = 8 per subgroup), 5000 cycles of thermal cycling, and a further 10,000 brushing cycles, with *R*_a_ and color measurements repeated after each step with the same equipment. Stain durability was assessed by calculating color differences (Δ*E*_00_) between consecutive time points and between baseline and final measurements (total) using the CIEDE2000 formula. One‐way analysis of variance and Tukey tests were used to compare the baseline *R*_a_ values of tested materials, while generalized linear model analysis was used to evaluate the effect of material type, toothpaste type, and time interval on *R*_a_ and Δ*E*_00_ (*α* = 0.05).

**Results:**

SM‐ZR had the lowest baseline *R*_a_ among the tested materials (*p* < 0.001). The three‐way interaction among main factors affected the *R*_a_ and Δ*E*_00_ values (*p* ≤ 0.041). SM‐ZR had lower *R*_a_ than SM‐LD at baseline and after thermal cycling with both toothpastes, as well as after the first brushing cycle with RT and the second brushing cycle with WT (*p* ≤ 0.025). It also had a lower *R*_a_ than AM‐ZR after thermal cycling and the second brushing with WT (*p* ≤ 0.016). After thermal cycling, RT‐brushed SM‐ZR had lower Δ*E*_00_ than RT‐brushed AM‐ZR and WT‐brushed SM‐ZR (*p* = 0.004). These specimens also had their lowest Δ*E*_00_ after thermal cycling (*p* < 0.001). RT‐brushed AM‐ZR and SM‐LD had lower Δ*E*_00_ after thermal cycling and after the second brushing than their total Δ*E*_00_ (*p* ≤ 0.027). After thermal cycling, Δ*E*_00_ of AM‐ZR and after the second brushing, Δ*E*_00_ of SM‐LD were lower than their total Δ*E*_00_ when brushed with WT (*p* ≤ 0.001).

**Conclusions:**

The *R*_a_ of AM‐ZR was comparable to that of SM‐ZR and SM‐LD at all time points, in terms of the previously reported clinical acceptability threshold. The color change for all materials after aging, including two cycles of brushing with toothpaste and thermal cycling, was moderately unacceptable considering previously published thresholds.

Dental ceramics are widely used in restorative dentistry, with zirconia favored for its high mechanical strength and esthetic properties, making it suitable for single‐ and multiple‐unit restorations.^1^, ^2^, ^3^, ^4^ Zirconia restorations are commonly fabricated using subtractive manufacturing, where dense prefabricated blocks are milled into the desired shape.^5^ However, milling has limitations, including material waste, difficulty producing complex internal geometries, and the risk of microcracks from tool vibration or wear.^6^, ^7^, ^8^, ^9^ Additive manufacturing offers a viable alternative by reducing material waste, enabling scalability, and allowing the fabrication of intricate geometries.^6^ Various additive manufacturing techniques have been proposed for zirconia,^10^, ^11^, ^12^, ^13^, ^14^ with digital light processing (DLP) being a prominent method,^15^ in which zirconia particles are suspended in a photopolymerizable resin and selectively polymerized layer by layer.^16^ Printed objects are subsequently debinded in a furnace to remove the organic binder and sintered at high temperatures to achieve the required density and mechanical properties.^17^, ^18^ Despite these advantages, the layer‐by‐layer fabrication process, together with the subsequent debinding and sintering steps, may introduce microstructural heterogeneity, residual porosity, and surface irregularities, which may influence the surface integrity and long‐term performance of additively manufactured zirconia restorations.^19^

Monolithic zirconia restorations often require additional measures to optimize esthetic outcomes.^20^, ^21^ Preshaded or multilayered zirconia disks with varying translucency, along with intrinsic or extrinsic staining, provide esthetically optimized options for subtractively manufactured zirconia.^5^, ^22^, ^23^, ^24^, ^25^ In contrast, esthetic customization for additively manufactured zirconia, particularly DLP‐printed restorations, remains limited due to the narrower shade and translucency range of available slurries. When debinding and sintering are performed separately, intrinsic staining is possible; however, combined firing protocols, which reduce overall firing time, only allow extrinsic staining. Although extrinsic staining achieves clinically acceptable esthetics,^26^ the long‐term stability of the applied stains may be affected by intraoral conditions such as temperature fluctuations and mechanical wear from brushing.^21^, ^27^, ^28^

Previous studies have investigated the surface and optical properties of additively manufactured zirconia;^20^, ^29^, ^30^, ^31^, ^32^ however, changes in surface roughness, which may influence plaque accumulation, antagonist wear, and the retention of extrinsic stains, as well as the durability of extrinsic stains, which is important for maintaining the long‐term esthetic appearance of restorations, remain insufficiently investigated. In particular, data evaluating how routine oral hygiene procedures with toothpastes of varying relative dentin abrasivity (RDA), combined with thermal aging, influence both the surface integrity and extrinsic stain durability of additively manufactured zirconia are lacking. With the increasing use of additively manufactured zirconia,^18^, ^33^ understanding how routine oral hygiene with toothpastes of varying RDA and thermal fluctuations affects its surface integrity and esthetic longevity is essential for predicting long‐term clinical performance. Therefore, this study aimed to evaluate the effects of brushing with different toothpastes and thermal aging on the surface roughness and stain durability of additively manufactured zirconia, compared with subtractively manufactured zirconia and lithium disilicate, the latter of which has shown high extrinsic stain durability under similar conditions.^26^, ^27^ The null hypotheses were that (i) material type would not affect the baseline surface roughness; (ii) material type, toothpaste type, and time interval (baseline, after first brushing, after thermal cycling, and after second brushing) would not affect the surface roughness; and (iii) material type, toothpaste type, and time interval (after first brushing, after thermal cycling, after second brushing, and after all tests are completed) would not affect the stain durability of tested ceramics.

## MATERIALS AND METHODS

Each subgroup (*n* = 8 per material‐toothpaste pair) was determined via a priori power analysis (*α* = 0.05, 1‐*β* = 0.90, effect size *f* = 0.90) based on a study with similar outcomes.^21^ A disk‐shaped file (Ø13 × 2 mm) was designed in standard tessellation language format using a design software program (Meshmixer v3.5.474; Autodesk Inc.) and imported into the nesting software (ZiproS v3.1.106; AON) of a DLP‐based printer (KUAIPRINT; Manfredi), where it was oriented horizontally on the build platform to fabricate the additively manufactured zirconia (AM‐ZR) specimens. Support structures were generated automatically, and the design was duplicated to place four specimens on the build platform. Specimens for each subgroup were fabricated in two separate printing jobs using a 3Y‐TZP zirconia slurry (INNI Cera BCM W1000; AON) with a layer thickness of 50 µm. After printing, specimens were ultrasonically cleaned in isopropyl alcohol for four minutes (2 min prewash, 2 min postwash) and air‐dried. AM‐ZR specimens then underwent a combined debinding and sintering process in a furnace (Zyntaprint‐S; Manfredi) lasting 21 h 45 min, with gradual heating to 1500°C and programmed holding stages at 150°C, 200°C, 320°C, and 490°C for complete debinding. Final sintering was performed at 1500°C for 2 h, followed by controlled cooling to 150°C. For subtractively manufactured zirconia (SM‐ZR) specimens, the disk‐shaped design file was imported into the nesting software (Ceramill Match 2; Amann Girrbach) of a five‐axis milling unit (Ceramill Motion 2; Amann Girrbach) and milled from a 4Y‐TZP zirconia disk (VITA YZ ST; Vita Zahnfabrik). After milling, specimens were sintered in a zirconia furnace (Ceramill Therm 3; Amann Girrbach) at a heating rate of 10°C/minute, held at 1550°C for 2 h, and cooled to room temperature at the same rate, following the manufacturer's instructions. Subtractively manufactured lithium disilicate (SM‐LD) specimens were milled from precrystallized lithium disilicate blocks (UP.CAD HT; Shenzhen Upcera Dental Technology) using the same five‐axis unit and then crystallized in a furnace (Programat P310; Ivoclar AG). The dimensions of all specimens were verified with a digital caliper (NB60; Mitutoyo American Corporation). Table 1 details the materials tested in this study.

**TABLE 1:** List of materials tested in this study.

	Manufacturing method	Chemical composition (wt%)
AM‐ZR	Additive manufacturing from a 3Y‐TZP zirconia slurry (INNI Cera BCM‐W1000)	ZrO_2_: 92.5% Y_2_O_2_: 4.67% HfO_2_: 1.64% SiO_2_: 0.58% SnO_2_: 0.15% Na_2_O: 0.14% MgO: 0.14% K_2_O: 0.07% Al_2_O_3_: 0.07%
SM‐ZR	Subtractive manufacturing from 4Y‐TZP disk (VITA YZ ST)	ZrO_2_: 88%–93% Y_2_O_3_: 6%–8% HfO_2_: 1%–3% Al_2_O_3_: 0%–1% Pigments: 0%–1%
SM‐LD	Subtractive manufacturing from lithium disilicate block (UP.CAD)	SiO_2_: 58.5–72.5% Li_2_O: 13–15% K_2_O: 3–5% Other oxides: 7.5–25%

Abbreviations: AM‐ZR, additively manufactured zirconia; SM‐LD, subtractively manufactured lithium disilicate; SM‐ZR, subtractively manufactured zirconia.

After fabrication, an external stain (B4, VITA AKZENT Plus; Vita Zahnfabrik) was applied to one surface of each specimen by attaching an applicator (Micro‐Stix; Microbrush International) to the opposite surface and dipping the specimen into the stain. No polishing was performed on the specimens prior to staining and glazing. Specimens were then fired in the same furnace used for SM‐LD crystallization. A glaze (VITA AKZENT Plus Glaze LT; Vita Zahnfabrik) was subsequently applied using the same dipping technique, followed by a second firing. All staining and glazing procedures were performed by one experienced prosthodontist (E.K.).

Following the staining and glazing procedures, baseline images of specimens were taken with a laser microscope (Keyence Laser Microscope VK‐X3000 Series; Keyence Corp) at ×20 magnification for topographical analysis. Then, the baseline *R*_a_ of the specimens was evaluated using a noncontact optical profilometer (FRT MicroProf 100, equipped with a CWL 300 µm sensor, resolution of 3 nm in *z*‐dimension; Fries Research & Technology GmbH) equipped with an analysis software program (Mark III; Fries Research & Technology GmbH). For each specimen, six linear measurements, three in vertical and three in horizontal direction, were obtained over 5.5‐mm‐long traces spaced 1 mm apart. Data were acquired at a resolution of 5501 points per line, using a cut‐off value of 0.8 mm.^34^ The arithmetic mean of these six measurements was calculated to determine the *R*_a_ value for all specimens.

After the *R*_a_ measurements, the color coordinates (L*, a*, and b*) of all specimens were measured three times against a gray background (L* = 52.14, a* = −1.23, b* = −3.44) using a digital spectrophotometer (CM‐26d; Konica Minolta) with an 8‐mm aperture. The device estimated the coordinates based on the Commission Internationale de l’Éclairage (CIE) Standard 2° observer characteristics. It was equipped with an integrated CIE D65 illuminant, and the spectral component was excluded during all measurements. A saturated sucrose solution (refractive index of approximately 1.5) was applied to ensure optimal optical contact between the specimens and the backing material.^35^ All measurements were conducted by one experienced prosthodontist (C.S.G.) in a room with controlled temperature, humidity, and natural light. Prior to measuring each subgroup, the spectrophotometer was calibrated, and the average of three measurements was calculated for each specimen.

**FIGURE 1:**
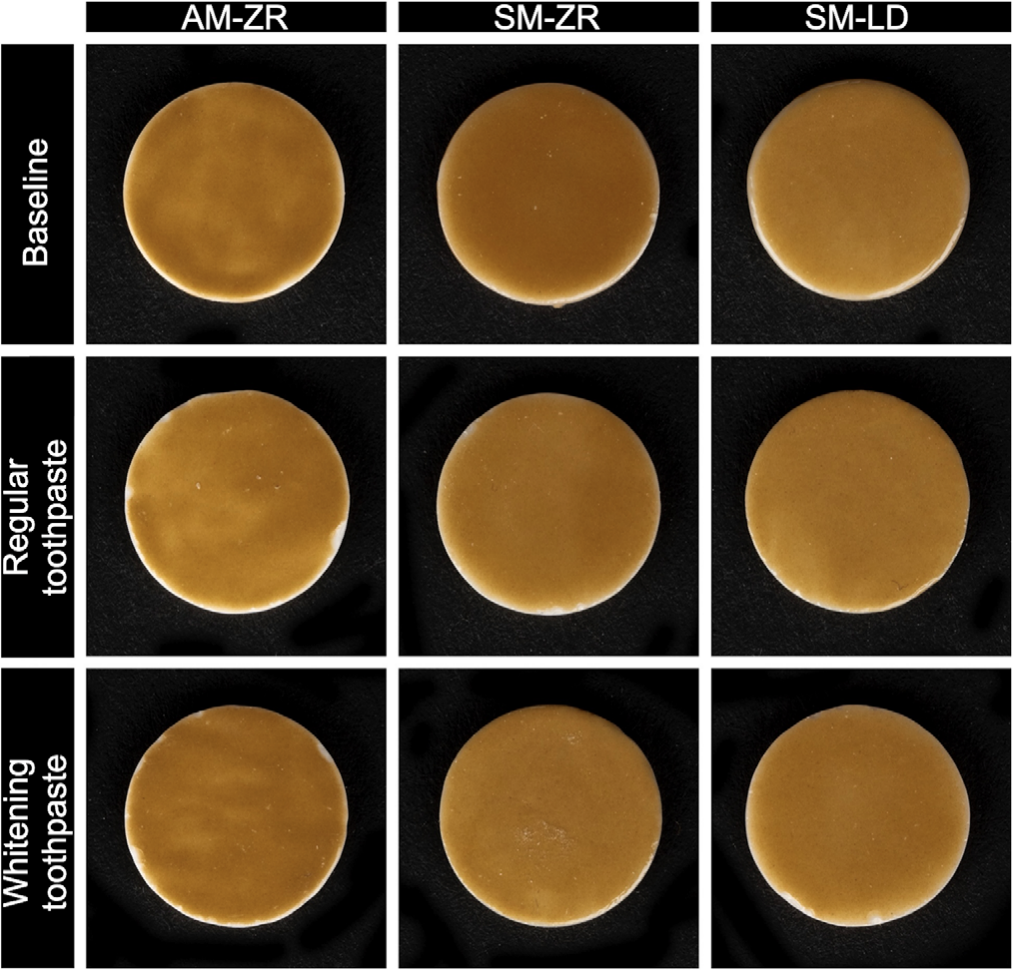
Representative images of specimens before and after procedures. AM‐ZR, additively manufactured zirconia; SM‐LD, subtractively manufactured lithium disilicate; SM‐ZR, subtractively manufactured zirconia.

**FIGURE 2:**
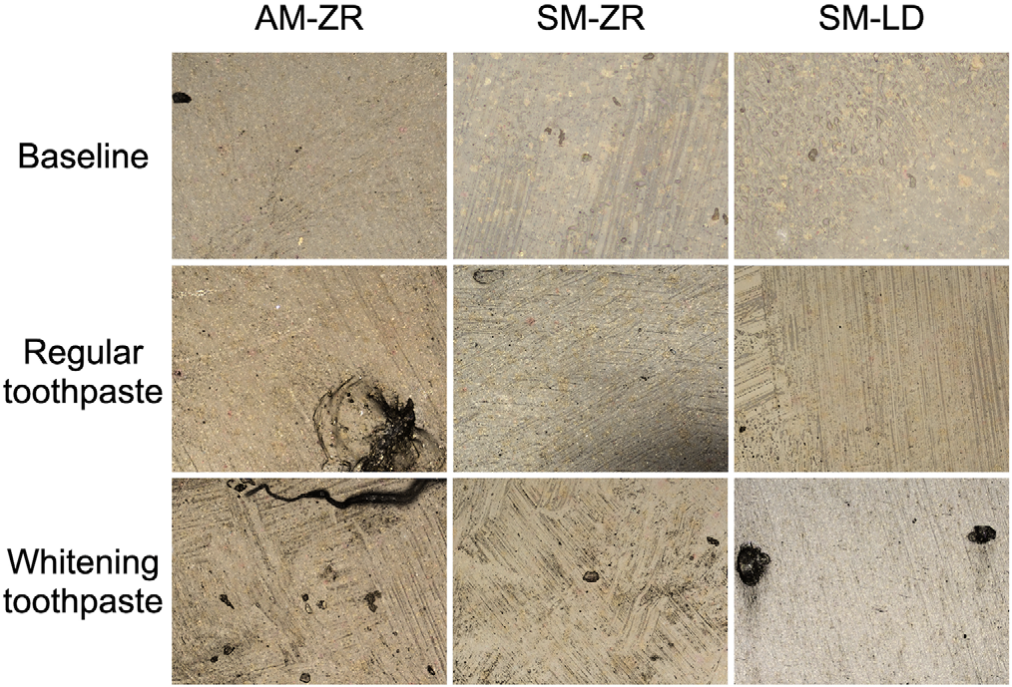
Representative laser microscope images (20×) of specimens before and after procedures. AM‐ZR, additively manufactured zirconia; SM‐LD, subtractively manufactured lithium disilicate; SM‐ZR, subtractively manufactured zirconia.

After baseline *R*_a_ and color measurements, all specimens underwent 5000 cycles of simulated brushing (10,000 strokes) using an automatic brushing machine operating at 1.5 Hz and Federal Drug Administration‐approved soft‐bristle toothbrushes (Oral‐B 40; Procter & Gamble).^36^ This regimen approximates 1 year of intraoral brushing, assuming a tooth is brushed approximately 20 times per day, totaling approximately 3650 cycles (7300 strokes) annually.^34^, ^37^ Two toothpaste slurries were prepared by mixing each toothpaste with deionized water in a 2:1 ratio by weight using a homogenizer (T25 digital Ultra Turrax; IKA).^27^ The first slurry was prepared using a regular toothpaste (Colgate Total CleanMint; Colgate‐Palmolive, RDA = 70, RT) and the second with a whitening toothpaste containing hydrogen peroxide (Colgate Optic White; Colgate‐Palmolive, RDA = 100, WT). Both slurries were freshly prepared and dispensed into the brushing machine chambers to ensure complete specimen coverage. During brushing, a vertical load of 200 g was applied while brushes moved horizontally at room temperature (23°C). After the first brushing cycle, specimens underwent 5000 thermal cycles between 5°C and 55°C in distilled water, with a 30‐s dwell time and 10‐s transfer time, simulating 6 months of intraoral use.^38^ This was followed by a second brushing cycle of 10,000 cycles (20,000 strokes), which represents 2 years of intraoral brushing, as previously described. During the second brushing cycle, both the slurries and toothbrushes were replaced after 5000 cycles (Figure 1).^39^, ^40^ After each process, the specimens were rinsed with distilled water, gently air‐dried, and the *R*_a_ and color coordinates were remeasured. The same microscope was then used to capture images of the specimens in their final state to observe the topographical changes (Figure 2). Color differences (Δ*E*_00_) between consecutive time intervals and between the baseline and final measurements (total) were calculated using the CIEDE2000 formula, with all parametric factors (KL, KC, and KH) set to 1,^34^, ^41^ to assess the stain durability of tested materials.

Normality of *R*_a_ and Δ*E*_00_ data was assessed using the Shapiro‐Wilk test. *R*_a_ data were normally distributed, while Δ*E*_00_ data required logarithmic (log) transformation to meet normality. Baseline *R*_a_ of the ceramics (*N* = 16 per material) was compared using one‐way analysis of variance and Tukey tests, whereas *R*_a_ and log‐transformed Δ*E*_00_ data (*n* = 8 per subgroup) were analyzed with a generalized linear model including material type, toothpaste type, and time interval as main factors, along with all possible interactions, with Bonferroni‐corrected post hoc comparisons. All analyses were performed with a statistical analysis software program (SPSS Statistics v30; IBM Corp), with significance set at *α* = 0.05. The perceptibility and acceptability of the Δ*E*_00_ values were further evaluated based on thresholds established in a previous study (not perceptible: ≤0.8, perceptible but clinically acceptable: ≤1.8, moderately unacceptable: ≤3.6, clearly unacceptable: ≤5.4, and extremely unacceptable: >5.4 units).^42^

## RESULTS

**FIGURE 3:**
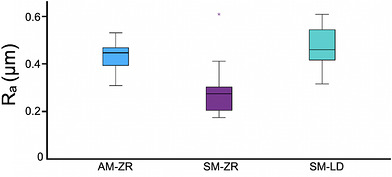
Box‐plot graph of baseline surface roughness values (µm). AM‐ZR, additively manufactured zirconia; SM‐LD, subtractively manufactured lithium disilicate; SM‐ZR, subtractively manufactured zirconia.

Significant differences were observed in the baseline *R*_a_ of tested ceramics (*p* < 0.001). SM‐ZR specimens had the lowest *R*_a_ among the tested ceramics (*p* < 0.001). However, the difference between AM‐ZR and SM‐LD specimens was nonsignificant (*p* = 0.391, Figure 3).

**FIGURE 4:**
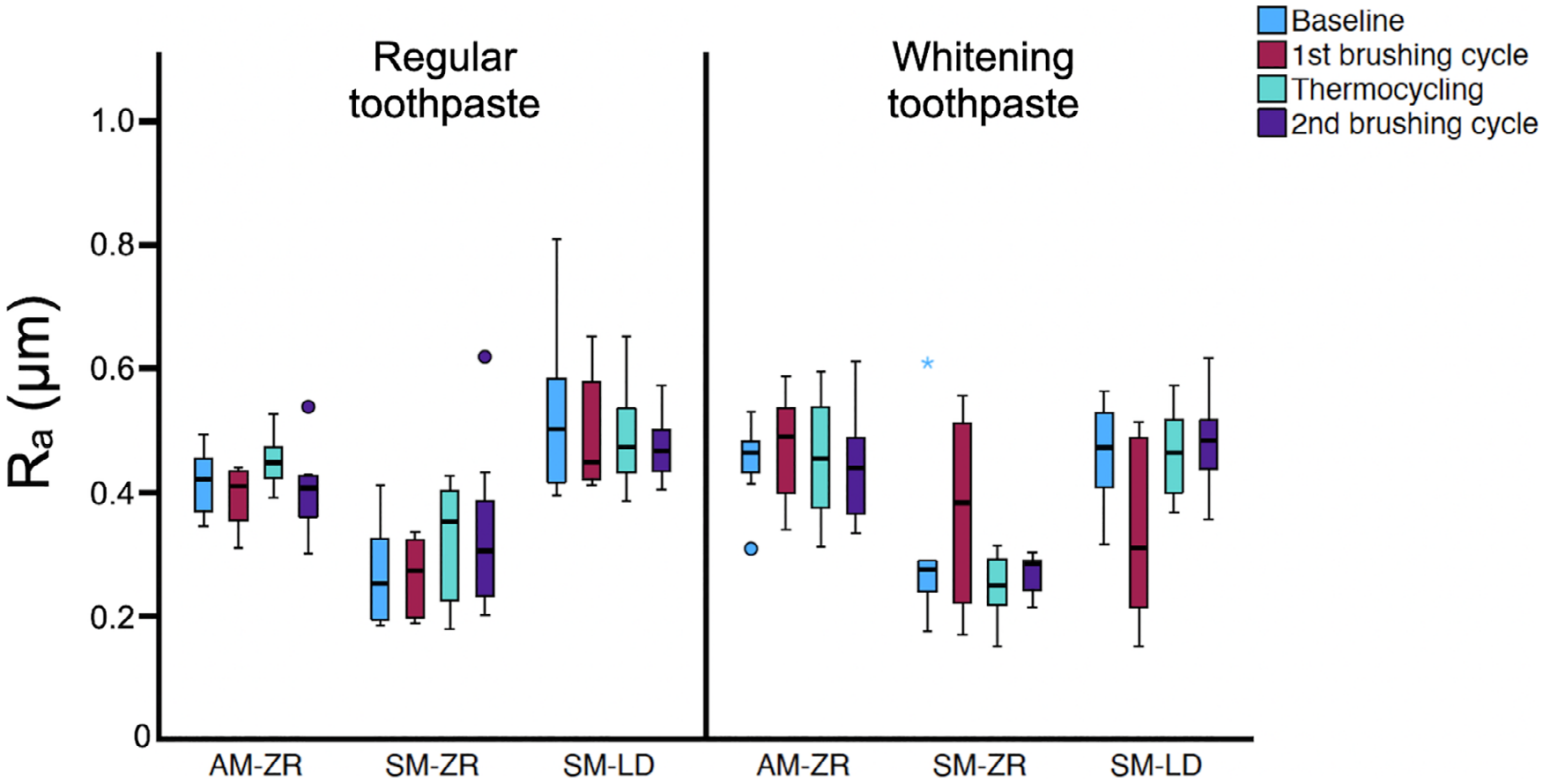
Box‐plot graph of surface roughness values of subgroups (µm). AM‐ZR, additively manufactured zirconia; SM‐LD, subtractively manufactured lithium disilicate; SM‐ZR, subtractively manufactured zirconia.

Table 2 presents the generalized linear model results, showing that interactions among all main factors influenced both *R*_a_ and Δ*E*_00_ (*p* ≤ 0.041). In clinically relevant comparisons of *R*_a_, SM‐ZR specimens showed lower values at baseline, after first brushing, and after thermal cycling than SM‐LD specimens when brushed with RT (*p* ≤ 0.025). When brushed with WT, SM‐ZR specimens showed lower *R*_a_ at baseline than SM‐LD specimens (*p* = 0.043), and lower values after thermal cycling and after the second brushing than AM‐ZR and SM‐LD specimens (*p* ≤ 0.016). For each material, no statistically significant differences were found across the tested time intervals, regardless of whether the specimens were brushed with the same or different toothpastes (*p* ≥ 0.064, Figure 4).

**TABLE 2:** Results of generalized linear model analysis.

	*R*_a_	Δ*E*_00_
Material	<0.001	0.001
Toothpaste	0.557	0.011
Time interval	0.790	<0.001
Material × toothpaste	0.005	0.141
Material × time interval	<0.001	0.018
Toothpaste × time interval	0.684	0.022
Material × toothpaste × time interval	0.003	0.041

Representative surface images (Figure 2) revealed differences in surface topography among the tested ceramics and brushing conditions. At baseline, SM‐ZR exhibited a relatively smoother and more uniform surface with fewer visible grooves compared with AM‐ZR and SM‐LD. After brushing with RT, all materials showed the appearance of brushing‐induced scratches, which were more pronounced on AM‐ZR and SM‐LD surfaces, while SM‐ZR maintained a comparatively more uniform topography. Brushing with WT resulted in more evident surface alterations, including deeper and more irregular grooves across all materials. These changes were particularly noticeable for AM‐ZR and SM‐LD specimens, whereas SM‐ZR surfaces still appeared relatively less affected.

**FIGURE 5:**
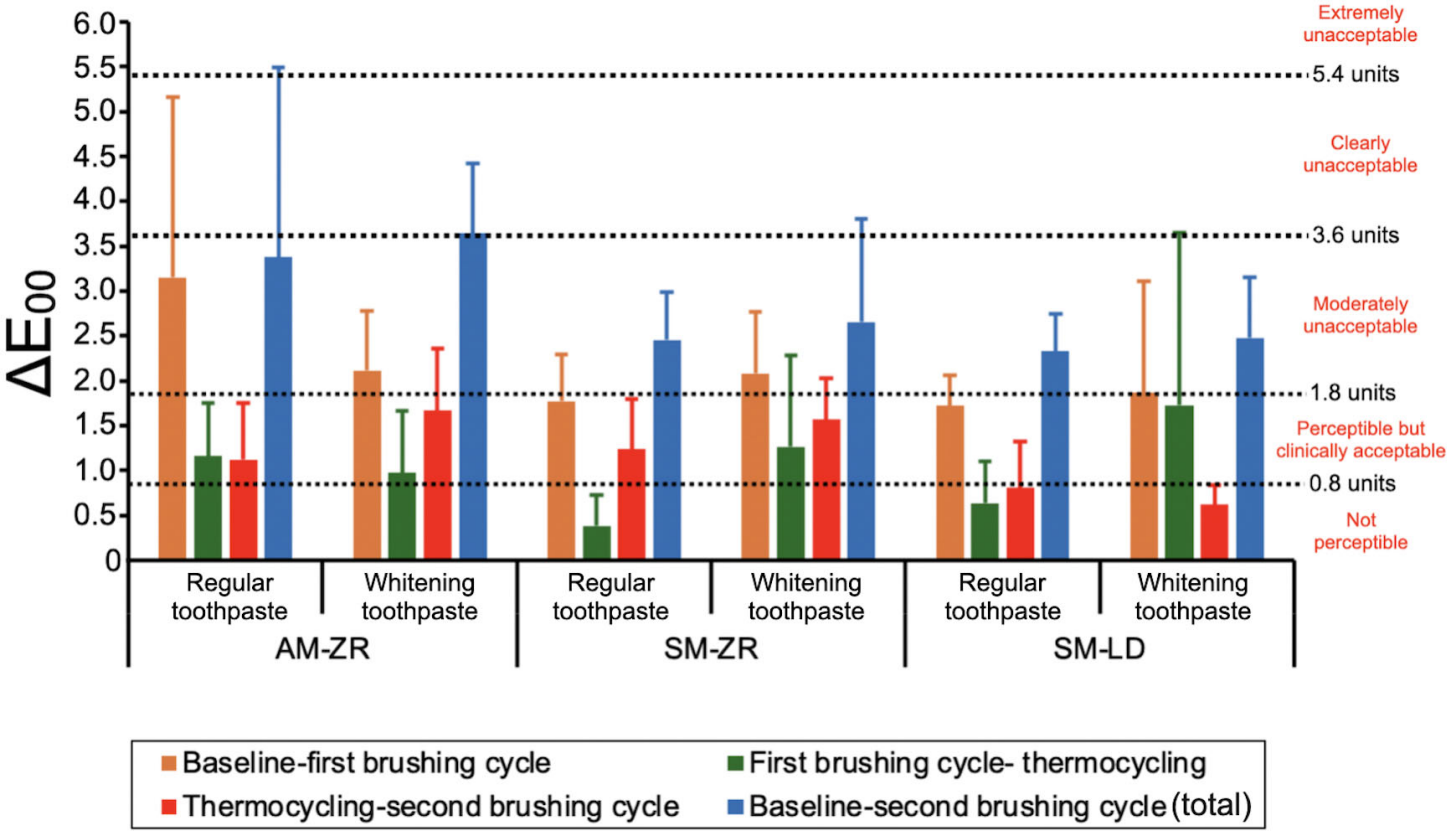
Δ*E*_00_ values of subgroups at each time interval. AM‐ZR, additively manufactured zirconia; SM‐LD, subtractively manufactured lithium disilicate; SM‐ZR, subtractively manufactured zirconia.

**FIGURE 6:**
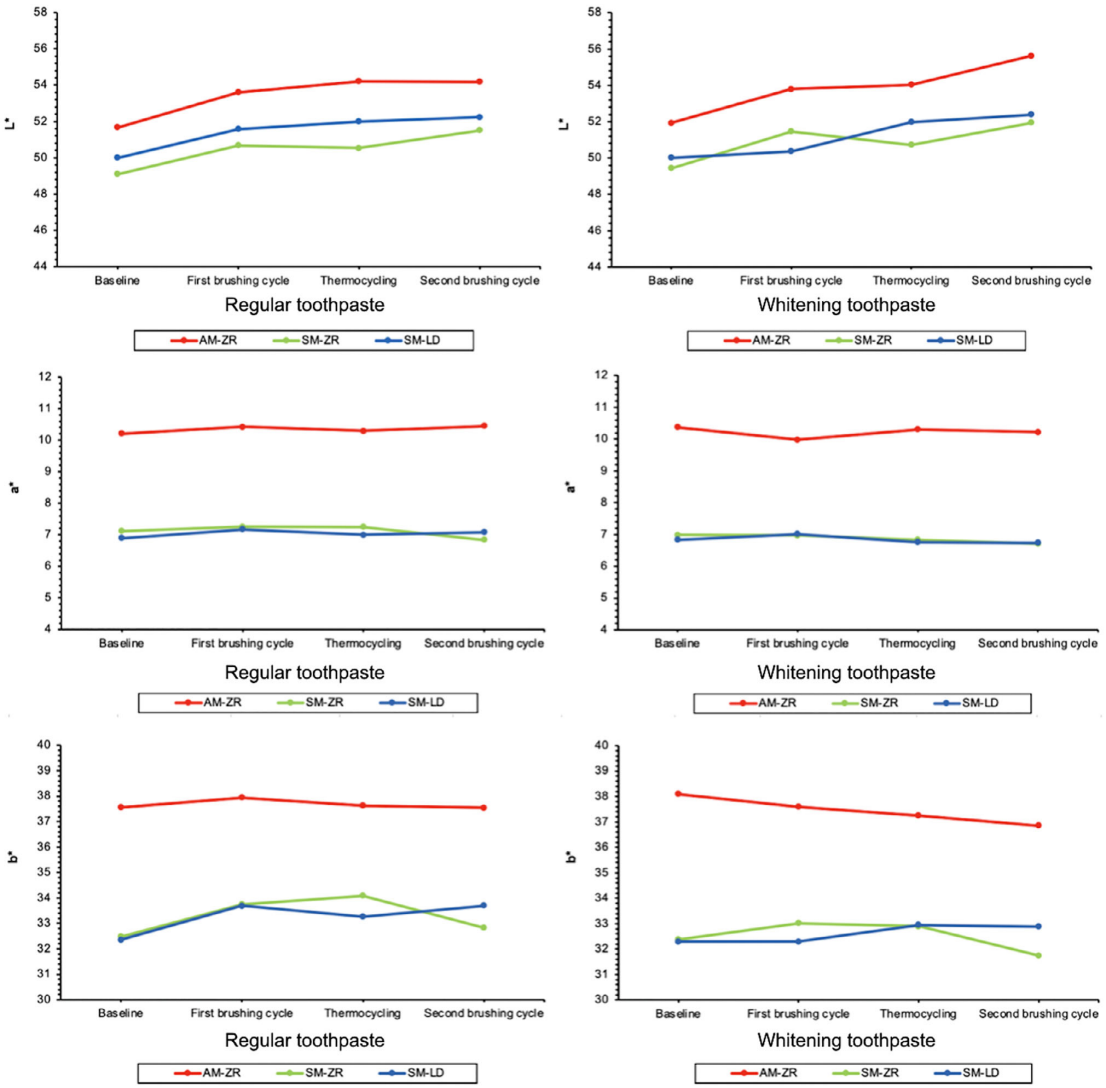
L*, a*, and b* values of tested specimens after each procedure. AM‐ZR, additively manufactured zirconia; SM‐LD, subtractively manufactured lithium disilicate; SM‐ZR, subtractively manufactured zirconia.

When clinically relevant comparisons of Δ*E*_00_ were evaluated, the only significant difference among tested materials was observed after thermal cycling, as SM‐ZR specimens had lower Δ*E*_00_ than AM‐ZR specimens when brushed with RT (*p* = 0.004). SM‐ZR specimens brushed with RT also had lower Δ*E*_00_ than those brushed with WT after thermal cycling (*p* = 0.004). After thermal cycling and after the second brushing, Δ*E*_00_ of AM‐ZR specimens brushed with RT was lower than their total Δ*E*_00_ (*p* ≤ 0.027), while SM‐ZR specimens brushed with RT had their lowest Δ*E*_00_ after thermal cycling (*p* < 0.001). After thermal cycling and after the second brushing, Δ*E*_00_ of SM‐LD specimens brushed with RT was lower than their total Δ*E*_00_ (*p* ≤ 0.002), while after the first brushing, Δ*E*_00_ was higher than after thermal cycling Δ*E*_00_ (*p* = 0.004). After thermal cycling, Δ*E*_00_ of AM‐ZR specimens brushed with WT was lower than their total Δ*E*_00_ (*p* = 0.001). In addition, after the second brushing, Δ*E*_00_ of SM‐LD specimens brushed with WT was lower than their total Δ*E*_00_ (*p* < 0.001, Table 3). Figure 5 presents the Δ*E*_00_ values of all subgroups in relation to the perceptibility and acceptability thresholds, while Figure 6 shows the trend in L*, a*, and b* value changes observed over the course of the procedures.

**TABLE 3:** Log‐transformed mean Δ*E*_00_ values and standard deviations (raw Δ*E*_00_ values and standard deviations).

		Material		
Toothpaste	Time interval	AM‐ZR	SM‐ZR	SM‐LD
Regular toothpaste	Baseline‐first brushing	0.41 ± 0.31^fgh^ (3.15 ± 2.00)	0.23 ± 0.13^defgh^ (1.78 ± 0.52)	0.23 ± 0.09^defgh^ (1.72 ± 0.34)
	First brushing‐thermal cycling	−0.01 ± 0.29^bcdef^ (1.16 ± 0.59)	−0.52 ± 0.31^a^ (0.39 ± 0.34)	−0.29 ± 0.30^abc^ (0.64 ± 0.47)
	Thermal cycling‐second brushing	−0.02 ± 0.28^bcdef^ (1.12 ± 0.63)	0.05 ± 0.21^bcdefg^ (1.24 ± 0.55)	−0.17 ± 0.29^abcd^ (0.81 ± 0.51)
	Baseline‐second brushing (total)	0.46 ± 0.26^gh^ (3.38 ± 2.12)	0.38 ± 0.09^fgh^ (2.46 ± 0.53)	0.36 ± 0.08^fgh^ (2.33 ± 0.41)
Whitening toothpaste	Baseline‐first brushing	0.30 ± 0.14^efgh^ (2.11 ± 0.67)	0.30 ± 0.15^efgh^ (2.08 ± 0.68)	0.20 ± 0.26^cdefgh^ (1.87 ± 1.24)
	First brushing‐thermal cycling	0.97 ± 0.69^abcde^ (3.15 ± 2.00)	0.00 ± 0.29^bcdef^ (1.27 ± 1.02)	0.06 ± 0.41^bcdefg^ (1.73 ± 1.93)
	Thermal cycling‐second brushing	0.14 ± 0.38^bcdefgh^ (1.67 ± 0.70)	0.18 ± 0.14^cdefgh^ (1.57 ± 0.45)	−0.23 ± 0.15^abc^ (0.62 ± 0.22)
	Baseline‐second brushing (total)	0.55 ± 0.10^h^ (3.64 ± 0.78)	0.38 ± 0.22^fgh^ (2.66 ± 1.15)	0.38 ± 0.13^fgh^ (2.48 ± 0.68)

*Note*: Different superscript lowercase letters indicate significant differences among material‐toothpaste‐time interval groups (*p *< 0.05).

Abbreviations: AM‐ZR, additively manufactured zirconia; SM‐LD, subtractively manufactured lithium disilicate; SM‐ZR, subtractively manufactured zirconia.

## DISCUSSION

The present study evaluated the effects of brushing with different toothpastes and thermal aging on the surface roughness and stain durability of additively manufactured zirconia, compared with subtractively manufactured zirconia and lithium disilicate. The null hypotheses were that the material type would not affect baseline *R*_a_, and that material type, toothpaste type, and time interval would not affect the *R*_a_ or stain durability of the tested ceramics. Material type significantly affected baseline *R*_a_ values, and the interaction among material type, toothpaste, and time interval significantly influenced both *R*_a_ and stain durability throughout the experiments. All null hypotheses were therefore rejected.

Specimens in this study were standardized for shade, finishing, and staining, and the differences in outcomes may be attributed to variations in ceramic composition. Baseline *R*_a_ values differed significantly among the ceramics, with SM‐ZR exhibiting lower *R*_a_ than AM‐ZR and SM‐LD, while no significant difference was observed between AM‐ZR and SM‐LD. These findings partly contrast with a previous report,^3^ although direct comparisons are limited by the absence of data on additively manufactured zirconia subjected to the same staining and glazing procedures. Habib et al.^3^ evaluated subtractively manufactured 3Y‐TZP, 4Y‐TZP, and lithium disilicate specimens stained and glazed with the same materials, and found no baseline differences between 3Y‐TZP and lithium disilicate, while 4Y‐TZP showed significantly higher values, which contrasts with the present results. The discrepancy may be due to differences in application techniques, as this study used dipping, whereas Habib et al.^3^ applied the materials with a brush, and the specific materials tested in each study.

Under humid conditions, zirconia may undergo low‐temperature degradation, where superficial tetragonal grains transform into the monoclinic phase, causing microcracks and rougher surfaces.^4^ Such phase transformation may alter surface topography and potentially influence stain uptake or retention, particularly in long‐term intraoral conditions. Although thermal cycling in this study could theoretically contribute to low‐temperature degradation, the relatively limited number of cycles and controlled temperature range potentially have minimized this effect. In this study, brushing with RT toothpaste revealed no significant differences in *R*_a_ between AM‐ZR and SM‐ZR at any aging time interval. Similarly, no differences were observed between AM‐ZR and SM‐LD. When WT toothpaste was used, AM‐ZR and SM‐LD also showed no significant *R*_a_ differences at any interval. Direct comparisons with SM‐ZR were more complex: SM‐ZR showed lower *R*_a_ than SM‐LD at certain intervals in both toothpaste groups, while AM‐ZR had higher *R*_a_ than SM‐ZR when brushed with WT. The clinical relevance of these findings remains uncertain, as no statistically significant differences were detected within subgroups, regardless of the toothpaste, or between subgroups of the same material. Importantly, the mean *R*_a_ values for all materials, both at baseline and across all aging intervals with either toothpaste, were below 0.5 µm. This threshold, reported by Jones et al.^43^ and supported by subsequent studies,^21^, ^44^ represents the limit for in vivo lingual tactile detection of *R*_a_ in dental restorations. Therefore, the values obtained in this study may not be clinically perceivable when this threshold is considered.

The type and concentration of abrasive components in toothpaste can affect the *R*_a_ of restorative materials to varying degrees, depending on their RDA values.^21^, ^26^, ^43^ While whitening toothpastes have been shown to be more abrasive to resin composites,^45^, ^46^ studies on ceramics have reported mixed or even contradictory results.^25^, ^46^ In this study, the RT (RDA 70) and WT (RDA 100) toothpastes were chosen to represent the lower and upper bounds of the medium abrasiveness range, which may explain the lack of differences observed between toothpaste subgroups. However, direct comparison of the current *R*_a_ results with those from previous studies is limited, as the novel additively manufactured zirconia evaluated in this study has not been previously investigated.

In this study, color coordinates were measured against a gray background, which closely simulates the intraoral environment.^47^ All tested variables significantly influenced the Δ*E*_00_ values. However, from a clinical perspective, meaningful comparisons in stain durability were primarily observed between SM‐ZR and AM‐ZR specimens brushed with RT after thermal cycling, with SM‐ZR exhibiting significantly lower Δ*E*_00_. These findings, along with the relatively high standard deviations observed for AM‐ZR specimens, may reflect microstructural defects and heterogeneity inherent to the layer‐by‐layer fabrication process, which may increase variability in pigment retention and optical behavior; differences in microstructure associated with additive manufacturing, including potential variations in porosity, grain packing, sintered density, and surface topography, may also contribute to the observed differences in color stability between AM‐ZR and SM‐ZR specimens. SM‐ZR brushed with RT also showed lower Δ*E*_00_ than specimens brushed with WT after thermal cycling. Differences between aging intervals were generally insignificant, except for SM‐ZR with WT. It should be emphasized that statistical significance does not necessarily equate to clinical relevance, and clinically meaningful conclusions regarding the effects of study variables on Δ*E*_00_ require reference to established perceptibility and acceptability thresholds. In this study, Δ*E*_00_ values below 1.8 units were considered clinically acceptable.^42^ Values exceeding this threshold represent color changes that are perceptible and potentially unacceptable to both clinicians and patients, indicating possible esthetic compromise of restorations over time, although such changes do not necessarily imply functional failure. In the RT subgroup, SM‐ZR and SM‐LD specimens showed clinically acceptable color change after the first brushing (Δ*E*_00_ ≤ 1.78 units), whereas AM‐ZR displayed moderately unacceptable changes (Δ*E*_00_ = 3.15 units). After thermal cycling and a second brushing cycle, Δ*E*_00_ values were ≤1.24 units for all materials. However, after completing all procedures, specimens brushed with RT exhibited Δ*E*_00_ values ranging from 2.33 to 3.38 units, indicating moderately unacceptable color changes. In the WT subgroup, color changes for all materials exceeded the clinically acceptable threshold (1.87 ≤ Δ*E*_00_ ≤ 2.11 units) after the first brushing cycle. Subsequent thermal cycling and a second brushing cycle resulted in Δ*E*_00_ values within the clinically acceptable range for all materials (Δ*E*_00_ ≤ 1.73 units), except for AM‐ZR after thermal cycling (Δ*E*_00_ = 3.15 units). The total color change was moderately unacceptable for SM‐ZR and SM‐LD specimens (2.48 ≤ Δ*E*_00_ ≤ 2.66 units) and clearly unacceptable for AM‐ZR (Δ*E*_00_ = 3.64 units). Although Δ*E*_00_ values after the first brushing cycle were near or slightly above the clinically acceptable threshold, cumulative effects over 3 years of simulated brushing led to a progressive increase in Δ*E*_00_, exceeding the moderately unacceptable limit. This color change may be partly attributed to the dark staining shade used, as reflected by the gradual increase in lightness (L*) across consecutive aging intervals. Previous studies have reported clinically acceptable color changes for subtractively manufactured zirconia and lithium disilicate specimens after 5 years of simulated brushing with regular, abrasive, or whitening toothpastes, although the staining shades differed from those used in this study.^26^, ^27^

The aging protocol did not include natural salivary components or dietary chemicals, and occlusal or masticatory forces were not simulated. In addition, no surface polishing was performed prior to staining and glazing in order to reflect a clinically relevant workflow; however, this may have influenced baseline *R*_a_ and the interaction with extrinsic stains, as well as their resistance to brushing and thermal aging. The use of flat, standardized specimens does not replicate the complex morphology of clinical restorations. Only one shade per material and one staining paste were used, and each material was represented by a single brand. A universal stain and glaze system was applied, as manufacturer‐specific staining materials are currently unavailable for the additively manufactured zirconia used in this study. These factors may influence stain durability and limit the generalizability of the findings to other materials, brands, and staining systems. In addition, the additively manufactured zirconia consisted of 3Y‐TZP, whereas the subtractively manufactured zirconia consisted of 4Y‐TZP, as the additive manufacturing system used in this study is currently limited to 3Y‐TZP formulations. Differences in yttria content and microstructure between these zirconia types may influence surface‐related properties, including grain structure and phase composition, which could affect *R*_a_ and the interaction with extrinsic stains, as well as their resistance to brushing and thermal aging. Therefore, this should be considered a potential confounding factor when interpreting comparisons between manufacturing approaches. The brushing simulation was based on previously reported vertical loading parameters; however, the absence of standardized brushing protocols represents an additional limitation. Moreover, the combined effects of brushing, thermal cycling, and chemical exposure may differ in vivo due to patient‐specific factors such as diet, saliva composition, and occlusal habits. Zirconia phase transformation was not evaluated; thermal cycling may induce tetragonal‐to‐monoclinic transformation, potentially affecting surface topography and optical properties. In addition, roughness was assessed using one parameter (*R*_a_), which does not fully capture peak‐valley morphology; inclusion of additional parameters could provide a more comprehensive characterization. Long‐term surface and color changes beyond the simulated period were also not assessed. These factors may contribute to progressive wear of the stain and glaze layer, potentially increasing surface roughness and compromising color stability over time. Future studies should incorporate standardized and reproducible brushing protocols, a broader range of materials, shades, and staining systems, and evaluation of low‐temperature degradation and phase transformation to enhance the clinical relevance and interpretability of the findings. In addition, the potential influence of intrinsic pigmentation in subtractively manufactured zirconia, compared with reliance on extrinsic staining in additively manufactured zirconia, warrants further investigation, as restorations relying primarily on extrinsic staining may be more susceptible to esthetic alterations following surface wear.

## CONCLUSIONS

Within the limitations of this in vitro study, considering previously reported roughness thresholds, the surface roughness of extrinsically stained additively manufactured zirconia was comparable to that of subtractively manufactured zirconia and lithium disilicate after simulated toothbrushing and thermal cycling equivalent to over 3 years, using toothpastes with RDA values up to 100. The color change of extrinsically stained additively manufactured zirconia was comparable to that of subtractively manufactured zirconia and lithium disilicate after all artificial aging procedures, with changes considered moderately unacceptable according to previously published clinical thresholds, regardless of the toothpaste used.

## CONFLICT OF INTEREST STATEMENT

The authors declare no conflicts of interest.

## Data Availability

The data that support the findings of this study are available from the corresponding author upon reasonable request.

## References

[1] Abualsaud R , Alalawi H . Fit, precision, and trueness of 3D‐printed zirconia crowns compared to milled counterparts. Dent J (Basel). 2022;10:215. 36421402 10.3390/dj10110215 PMC9689223

[2] Dewan H . Clinical effectiveness of 3D‐milled and 3D‐printed zirconia prosthesis‐a systematic review and meta‐analysis. Biomimetics (Basel). 2023;8:394. 37754145 10.3390/biomimetics8050394 PMC10526775

[3] Habib AW , Aboushelib MN , Habib NA . Effect of chemical aging on color stability and surface properties of stained all‐ceramic restorations. J Esthet Restor Dent. 2021;33:636–47. 33665948 10.1111/jerd.12719

[4] Pinelli LAP , Olbera ACG , Candido LM , Miotto LN , Antonio SG , Fais LMG . Effects of whitening dentifrice on yttria‐stabilized tetragonal zirconia polycrystal surfaces after simulating brushing. J Prosthet Dent. 2017;117:158–63. 27475916 10.1016/j.prosdent.2016.05.005

[5] Huang B , Chen M , Wang J , Zhang X . Advances in zirconia‐based dental materials: properties, classification, applications, and future prospects. J Dent. 2024;147:105111. 38866229 10.1016/j.jdent.2024.105111

[6] Lerner H , Nagy K , Pranno N , Zarone F , Admakin O , Mangano F . Trueness and precision of 3D‐printed versus milled monolithic zirconia crowns: an in vitro study. J Dent. 2021;113:103792. 34481929 10.1016/j.jdent.2021.103792

[7] Methani MM , Revilla‐León M , Zandinejad A . The potential of additive manufacturing technologies and their processing parameters for the fabrication of all‐ceramic crowns: a review. J Esthet Restor Dent. 2020;32:182–92. 31701629 10.1111/jerd.12535

[8] Silva S , Silva NRD , Santos J , Moreira FGG , Özcan M , Souza R . Accuracy, adaptation and margin quality of monolithic zirconia crowns fabricated by 3D printing versus subtractive manufacturing technique: a systematic review and meta‐analysis of in vitro studies. J Dent. 2024;147:105089. 38772449 10.1016/j.jdent.2024.105089

[9] Giugliano TS , Zhang Y , Janal MN , Lim CH , Smith RM , Choi M . In vitro comparison of physical characteristics of milled versus printed zirconia discs. J Prosthodont. 2024;33:891–8. 37776103 10.1111/jopr.13778 PMC10980599

[10] Wang W , Yu H , Liu Y , Jiang X , Gao B . Trueness analysis of zirconia crowns fabricated with 3‐dimensional printing. J Prosthet Dent. 2019;121:285–91. 30017167 10.1016/j.prosdent.2018.04.012

[11] Wang L , Yu H , Hao Z , Tang W , Dou R . Investigating the effect of solid loading on microstructure, mechanical properties, and translucency of highly translucent zirconia ceramics prepared via stereolithography‐based additive manufacturing. J Mech Behav Biomed Mater. 2023;144:105952. 37311296 10.1016/j.jmbbm.2023.105952

[12] Xiang D , Xu Y , Bai W , Lin H . Dental zirconia fabricated by stereolithography: accuracy, translucency and mechanical properties in different build orientations. Ceram Int. 2021;47:28837–47.

[13] Revilla‐León M , Methani MM , Morton D , Zandinejad A . Internal and marginal discrepancies associated with stereolithography (SLA) additively manufactured zirconia crowns. J Prosthet Dent. 2020;124:730–7. 31980204 10.1016/j.prosdent.2019.09.018

[14] Teegen IS , Schadte P , Wille S , Adelung R , Siebert L , Kern M . Comparison of properties and cost efficiency of zirconia processed by DIW printing, casting and CAD/CAM‐milling. Dent Mater. 2023;39:669–76. 37230861 10.1016/j.dental.2023.05.001

[15] Demirel M , Diken Türksayar AA , Donmez MB , Schimmel M , Yilmaz B . Evaluation of the fabrication trueness and internal fit of additively manufactured two‐piece zirconia abutments with different build orientations compared to subtractively manufactured abutments. J Dent. 2025;153:105470. 39566714 10.1016/j.jdent.2024.105470

[16] Meng J , Lian Q , Xi S , Yi Y , Lu Y , Wu G . Crown fit and dimensional accuracy of zirconia fixed crowns based on the digital light processing technology. Ceram Int. 2022;48:17852–63.

[17] Sim J‐H , Koo B‐K , Jung M , Kim D‐S . Study on debinding and sintering processes for ceramics fabricated using digital light processing (DLP) 3D printing. Processes. 2022;10:2467.

[18] Alghauli M , Alqutaibi AY , Wille S , Kern M . 3D‐printed versus conventionally milled zirconia for dental clinical applications: trueness, precision, accuracy, biological and esthetic aspects. J Dent. 2024;144:104925. 38471580 10.1016/j.jdent.2024.104925

[19] Branco AC , Colaço R , Figueiredo‐Pina CG , Serro AP . Recent advances on 3D‐printed zirconia‐based dental materials: a review. Materials (Basel). 2023;16(5):1860. 36902976 10.3390/ma16051860 PMC10004380

[20] Cui X , Shen Z , Wang X . Esthetic appearances of anatomic contour zirconia crowns made by additive wet deposition and subtractive dry milling: a self‐controlled clinical trial. J Prosthet Dent. 2020;123:442–8. 31307809 10.1016/j.prosdent.2019.02.016

[21] Bayestehtarat S , Gullard A , Morrow B , Hollis W , Ragain J . Longevity of extrinsic stains on monolithic zirconia restorations: an in vitro study. J Prosthet Dent. 2023;130:877.e1–7. 10.1016/j.prosdent.2023.09.008 37845115

[22] Alqutaibi AY , Ghulam O , Krsoum M , Binmahmoud S , Taher H , Elmalky W , et al. Revolution of current dental zirconia: a comprehensive review. Molecules. 2022;27:1699. 35268800 10.3390/molecules27051699 PMC8911694

[23] Abounassif FM , Alfaraj A , Gadah T , Yang CC , Chu TG , Lin WS . Color stability of precolored and extrinsically colored monolithic multilayered polychromatic zirconia: effects of surface finishing and aging. J Prosthodont. 2026;35:169–75. 38923252 10.1111/jopr.13875 PMC12906326

[24] Silva S , Silva B , Crispim AHT , Dal Piva AMO , Kleverlaan CJ , Souza R . Effect of different finishing/polishing techniques and glaze application on the flexural strength of ultratranslucent zirconia after hydrothermal aging. J Mech Behav Biomed Mater. 2025;164:106924. 39923467 10.1016/j.jmbbm.2025.106924

[25] Sahadi BO , Domingues BC , Soto‐Montero J , de Araújo Neto VG , Riquieri H , Giannini M . Effect of toothbrushing on surface roughness, gloss, and topography of polished and glazed ultra‐translucent zirconia. J Prosthodont. 2024;33:103–9. 38526464 10.1111/jopr.13849

[26] Mascaro BA , Salomon J‐P , Demartine MS , Nicola TC , dos Santos Nunes Reis JM . Evaluation of color and translucency of stained and glazed monolithic lithium disilicates and zirconia after toothbrushing with different dentifrices and thermocycling. Int J Prosthodont. 2024;37:423–31. 37988426 10.11607/ijp.8495

[27] Sulaiman TA , Camino RN , Cook R , Delgado AJ , Roulet JF , Clark WA . Time‐lasting ceramic stains and glaze: a toothbrush simulation study. J Esthet Restor Dent. 2020;32:581–5. 32352643 10.1111/jerd.12590

[28] Yuan JC‐C , Barão VAR , Wee AG , Alfaro MF , Afshari FS , Sukotjo C . Effect of brushing and thermocycling on the shade and surface roughness of CAD‐CAM ceramic restorations. J Prosthet Dent. 2018;119:1000–6. 28965682 10.1016/j.prosdent.2017.06.001

[29] Zhu Z , Huang X , Lyu J , Yang X , Tan J , Liu X . Optical properties of monolithic zirconia fabricated with nanoparticle jetting. J Prosthet Dent. 2024;132:464.e1–8. 10.1016/j.prosdent.2024.05.005 38796354

[30] Abualsaud R , Abussaud M , Assudmi Y , Aljoaib G , Khaled A , Alalawi H , et al. Physiomechanical and surface characteristics of 3D‐printed zirconia: an in vitro study. Materials (Basel). 2022;15:6988. 36234329 10.3390/ma15196988 PMC9572578

[31] Zandinejad A , Khanlar LN , Barmak AB , Tagami J , Revilla‐León M . Surface roughness and bond strength of resin composite to additively manufactured zirconia with different porosities. J Prosthodont. 2022;31:97–104. 35313023 10.1111/jopr.13434

[32] Baysal N , Tuğba Kalyoncuoğlu Ü , Ayyıldız S . Mechanical properties and bond strength of additively manufactured and milled dental zirconia: a pilot study. J Prosthodont. 2022;31:629–34. 34940979 10.1111/jopr.13472

[33] Alghauli MA , Alqutaibi AY , Wille S , Kern M . The physical‐mechanical properties of 3D‐printed versus conventional milled zirconia for dental clinical applications: a systematic review with meta‐analysis. J Mech Behav Biomed Mater. 2024;156:106601. 38810545 10.1016/j.jmbbm.2024.106601

[34] Çakmak G , Donmez MB , de Paula MS , Akay C , Fonseca M , Kahveci Ç , et al. Surface roughness, optical properties, and microhardness of additively and subtractively manufactured CAD‐CAM materials after brushing and coffee thermal cycling. J Prosthodont. 2025;34:68–77. 37947220 10.1111/jopr.13796 PMC11729847

[35] Pecho OE , Ghinea R , Ionescu AM , de la Cruz Cardona J , Paravina RD , del Mar Pérez M . Color and translucency of zirconia ceramics, human dentine and bovine dentine. J Dent. 2012;40:e34–40. 22960460 10.1016/j.jdent.2012.08.018

[36] Wierichs RJ , Kogel J , Lausch J , Esteves‐Oliveira M , Meyer‐Lueckel H . Effects of self‐assembling peptide p11‐4, fluorides, and caries infiltration on artificial enamel caries lesions in vitro. Caries Res. 2017;51:451–9. 28772269 10.1159/000477215

[37] da Cruz MEM , Oliveira JJR , Dovigo LN , Fonseca RG . Long‐term effect of gastric juice alternating with brushing on the surface roughness, topography, and staining susceptibility of CAD‐CAM monolithic materials. J Prosthet Dent. 2022;127:659.e1–11. 10.1016/j.prosdent.2022.01.016 35184887

[38] Gale MS , Darvell BW . Thermal cycling procedures for laboratory testing of dental restorations. J Dent. 1999;27:89–99. 10071465 10.1016/s0300-5712(98)00037-2

[39] Mahrous AA , Alhammad A , Alqahtani F , Aljar Y , Alkadi A , Taymour N , et al. The toothbrushing effects on surface properties and color stability of CAD/CAM and pressable ceramic fixed restorations‐an in vitro study. Materials (Basel). 2023;16:2950. 37109785 10.3390/ma16082950 PMC10142931

[40] de Oliveira Dal Piva AM , Bottino MA , Anami LC , Werner A , Kleverlaan CJ , Lo Giudice R , et al. Toothbrushing wear resistance of stained CAD/CAM ceramics. Coatings. 2021;11:224.

[41] Çakmak G , Oosterveen‐Rüegsegger AL , Akay C , Schimmel M , Yilmaz B , Donmez MB . Influence of polishing technique and coffee thermal cycling on the surface roughness and color stability of additively and subtractively manufactured resins used for definitive restorations. J Prosthodont. 2024;33:467–74. 37421940 10.1111/jopr.13730

[42] Paravina RD , Pérez MM , Ghinea R . Acceptability and perceptibility thresholds in dentistry: a comprehensive review of clinical and research applications. J Esthet Restor Dent. 2019;31:103–12. 30891913 10.1111/jerd.12465

[43] Jones C , Billington R , Pearson G . The in vivo perception of roughness of restorations. Br Dent J. 2004;196:42–5. 14966503 10.1038/sj.bdj.4810881

[44] Da Costa J , Adams‐Belusko A , Riley K , Ferracane JL . The effect of various dentifrices on surface roughness and gloss of resin composites. J Dent. 2010;38:e123–8. 20193728 10.1016/j.jdent.2010.02.005

[45] Di Fiore A , Stellini E , Basilicata M , Bollero P , Monaco C . Effect of toothpaste on the surface roughness of the resin‐contained CAD/CAM dental materials: a systematic review. J Clin Med. 2022;11:767. 35160219 10.3390/jcm11030767 PMC8836682

[46] Mörmann WH , Stawarczyk B , Ender A , Sener B , Attin T , Mehl A . Wear characteristics of current aesthetic dental restorative CAD/CAM materials: two‐body wear, gloss retention, roughness and Martens hardness. J Mech Behav Biomed Mater. 2013;20:113–25. 23455168 10.1016/j.jmbbm.2013.01.003

[47] Ardu S , Braut V , Di Bella E , Lefever D . Influence of background on natural tooth colour coordinates: an in vivo evaluation. Odontology. 2014;102:267–71. 23934087 10.1007/s10266-013-0126-1

